# In Silico and In Vitro Tailoring of a Chitosan Nanoformulation of a Human Metabolic Enzyme

**DOI:** 10.3390/pharmaceutics13030329

**Published:** 2021-03-04

**Authors:** Paulo R. Lino, João Leandro, Mariana Amaro, Lídia M. D. Gonçalves, Paula Leandro, António J. Almeida

**Affiliations:** Faculty of Pharmacy, Research Institute for Medicines, Universidade de Lisboa, 1649-003 Lisboa, Portugal; proquelino@ff.ulisboa.pt (P.R.L.); jptleandro@gmail.com (J.L.); m.amaro@campus.fct.unl.pt (M.A.); lgoncalves@ff.ulisboa.pt (L.M.D.G.)

**Keywords:** chitosan, molecular modelling, enzyme therapeutics, self-assembly, nanoencapsulation, human phenylalanine hydroxylase

## Abstract

Enzyme nanoencapsulation holds an enormous potential to develop new therapeutic approaches to a large set of human pathologies including cancer, infectious diseases and inherited metabolic disorders. However, enzyme formulation has been limited by the need to maintain the catalytic function, which is governed by protein conformation. Herein we report the rational design of a delivery system based on chitosan for effective encapsulation of a functionally and structurally complex human metabolic enzyme through ionic gelation with tripolyphosphate. The rationale was to use a mild methodology to entrap the multimeric multidomain 200 kDa human phenylalanine hydroxylase (hPAH) in a polyol-like matrix that would allow an efficient maintenance of protein structure and function, avoiding formulation stress conditions. Through an in silico and in vitro based development, the particulate system was optimized with modulation of nanomaterials protonation status, polymer, counterion and protein ratios, taking into account particle size, polydispersity index, surface charge, particle yield production, protein free energy of folding, electrostatic surface potential, charge, encapsulation efficiency, loading capacity and transmission electron microscopy morphology. Evaluation of the thermal stability, substrate binding profile, relative enzymatic activity, and substrate activation ratio of the encapsulated hPAH suggests that the formulation procedure does not affect protein stability, allowing an effective maintenance of hPAH biological function. Hence, this study provides an important framework for an enzyme formulation process.

## 1. Introduction

The advent of recombinant DNA technology revolutionized the therapeutic approaches to a vast number of unmet medical disorders by boosting the development of new biologic pharmaceutical entities, including peptide and proteins [1]. According to their function and biomedical application, protein therapeutics have been grouped in four classes [2]: protein therapeutics with enzymatic or regulatory activity (Group I); protein therapeutics with special targeting activity (Group II); protein vaccines (Group III); and protein diagnostics (Group IV). Group I is particularly interesting as it includes enzymes that can be used to replace an enzyme that is deficient or abnormal (e.g., α-glucocerebrosidase for the inherited metabolic disorder Gaucher disease), to augment existing pathways (e.g., trypsin) or to provide a novel function or activity (e.g., L-asparaginase for acute lymphocytic leukemia). Although regarded as potent therapeutic agents, due to the high specificity and affinity for the pathogenic target (the enzyme’s substrate), development of effective and stable formulations has been hampered by the intrinsic conformational instability of these proteins. In fact, due to their role as catalysts of chemical reactions, enzyme structure must be particularly dynamic, greatly contributing to limited physicochemical stability during formulation, low circulation half-lives and propensity to be immunogenic. An effective strategy to overcome these constraints is based on innovative techniques of protein encapsulation with biopolymers such as chitosan (CS). Since 1989, an exponential increase occurred in the development of positively charged polysaccharide nanostructured delivery systems based on CS [3,4]. Obtained from the N-deacetylation of chitin, CS presents a wide bioavailability, cationic hydrophilic nature and is considered as biocompatible, biodegradable and extremely versatile regarding its hydrodynamic properties. Within each polymeric chain, the CS glucosamine backbone exhibits a rich density of both hydroxyl and amine groups that favor the interaction with polyanions and negatively charged macromolecules. Such features render it a viable raw material to develop delivery systems intended for labile active pharmaceutical ingredients (API). Nevertheless, CS application to the formulation of therapeutic proteins has been focused on smaller or less complex polypeptides (e.g., insulin and calcitonin), model protein drugs (e.g., bovine serum albumin and ovalbumin), and antigens (e.g., tetanus toxoid, diphtheria toxoid, recombinant hepatitis B surface antigen and *S. equi* antigens) either within an oncologic or infectious disease context [5,6,7,8,9]. Regarding enzyme immobilization in polymeric matrices, it has been mainly associated with applications to industrial processes (e.g., biocatalysis reactors, biosensors) and usually within a micrometric range of sizes [10,11,12,13], while some work has also been performed involving therapeutic enzymes, either in lipid-based [14,15,16,17] or chitosan-based nanoparticles [18]. It is expected that as the complexity of the protein structure and function increases towards multi-subunit, high molecular mass and fine-tuned functional regulation, enzyme encapsulation will be even more challenging. In recent years, in silico approaches have been demonstrated to be a useful platform in the prediction of chitosan/drug interactions, providing a valuable tool in the development of chitosan-based nanoparticles [19,20].

Herein is reported the development of a delivery system based on CS, to entrap human phenylalanine hydroxylase (hPAH) through an optimized ionic gelation with the polyanion tripolyphosphate (TPP), combining both in silico and experimental data. Human PAH is a hepatic enzyme responsible for phenylketonuria (PKU), the most frequent inherited disorder of amino acid metabolism. This enzyme hydroxylates l-phenylalanine (l-Phe) into l-tyrosine (l-Tyr) in the presence of the cofactor tetrahydrobiopterin (BH_4_) and dioxygen. The hPAH is a complex 200 kDa homotetrameric (dimer of dimers) non-heme iron enzyme, with each monomer presenting a regulatory, a catalytic and an oligomerization domain. The hPAH oligomeric and multidomain arrangement allows a complex regulation of enzyme activity, necessary for its biological function, including: (i) activation by its own substrate by l-Phe binding to the N-terminal regulatory domain; (ii) positive cooperative towards l-Phe and; (iii) inhibition by the cofactor BH_4_ [21]. Mechanistically, the complex hPAH regulation is a result of the protein structural flexibility that allows large molecular motions that also contribute to the reported protein instability [22]. In particular, the mobile N-terminal regulatory domain controls the response to the substrate as when it lays over the catalytic domain it sterically blocks the access of l-Phe to the active site, rendering a protein with a lower catalytic activity (resting state). The movement of the regulatory domains brings together two N-terminal domains from two different dimers, forming an interface for allosteric binding of l-Phe and leaving the catalytic center exposed to the solvents (activated state) [23,24,25].

The rationale was to entrap this flexible 200 kDa tetrameric enzyme in a stable polyol-like nanoparticulate matrix at a particle size suitable for long circulating parenteral delivery (≤200 nm), allowing an efficient maintenance of the intricate protein structure and function, avoiding the use of organic solvents, high shear stress and high temperature [26]. By using in silico and in vitro experimental approaches, a mechanistic overview of the critical steps involved in the design of the nanoparticulate drug delivery system was obtained.

## 2. Materials and Methods

### 2.1. Materials

*Escherichia coli* (*E. coli*) TOP 10, the prokaryotic expression vector pTrcHis and the fluorescent dye SYPRO orange (5000× stock concentration) were obtained from Invitrogen (Carlsbad, CA, USA). The cofactor BH_4_, l-Phe, catalase, Hepes, dithiothreitol (DTT), low molecular weight (MW) CS (MW: 61 kDa; viscosity: 42 cps) with a deacetylation degree (DD) of 91.8%, TPP and cibacron brilliant red 3B-A were from Sigma Chemical Co (St. Louis, MO, USA). Glycerol (molecular biology grade) was purchased from Merck KGaA (Darmstadt, Germany). All reagents were of analytical grade or equivalent.

### 2.2. Expression and Purification of Recombinant hPAH Tetramers

The hPAH recombinant protein was expressed in *E. coli* as fusion protein as previously described [27]. Cells were grown at 37 °C and expression was induced by the addition of 1 mM isopropyl-β-d-thiogalactoside (IPTG). Simultaneously, 0.2 mM ferrous ammonium sulphate was added to the culture medium. Bacteria were harvested 3 h after induction and disrupted by sonication. After centrifugation at 13,000× *g* for 40 min, at 4 °C, a soluble (supernatant) and insoluble fraction (pellet) was obtained. The soluble fraction was used to purify the fusion protein by immobilized metal affinity chromatography using a Ni-chelating resin (Qiagen, Valencia, CA, USA). Recombinant proteins were eluted with 250 mM imidazole in a phosphate buffer, as previously reported [28].

The recombinant tetramers were further purified by size exclusion chromatography (SEC), using a 1.6 cm × 60 cm HiLoad Superdex 200 HR column (GE-Healthcare Life Sciences; Uppsala, Sweden) and a mobile phase containing 20 mM Na-Hepes, 200 mM NaCl, pH 7.0 (SEC buffer) pumped at a flow rate of 0.7 mL/min (Figure S1, Supplementary Materials) [29]. The relative molecular mass of the different oligomeric forms was estimated from a calibration curve obtained with standard proteins (Figure S2): cytochrome C (12.4 kDa), myoglobin (17.6 kDa), ovalbumin (45 kDa), bovine serum albumin (BSA; 66kDa), alcohol dehydrogenase (150 kDa), β-amylase (200 kDa) and apoferritin (443 kDa). Blue Dextran 2000 and L-Tyr were used to determine the void volume (V_0_ = 46.6 mL) and the total exclusion volume (V_T_ = 119.4 mL) of the column, respectively. All purification steps were carried out at 4 °C. The tetrameric fusion protein was concentrated using an Amicon Ultra 15 centrifugal filter (MW cut-off of 30 kDa; Millipore; Billerica, MA, USA).

### 2.3. Preparation of CS Nanoparticles

The particulate system was obtained by ionic gelation using a slight modification of a previously described method [7,9]. Briefly, CS stock solution was prepared at 10 mg/mL in 1% (*v*/*v*) acetic acid and TPP stock solution at 10 mg/mL in ultra-pure water. Stock solutions were individually diluted to the desired concentrations (TPP diluted in SEC buffer). TPP was added to the CS solution, and the mixture was homogenized by gentle up-and-down pipetting, at RT. Nanoparticles were instantly formed when TPP was added to CS at a fixed 1:5 volume ratio.

For the preparation of protein-loaded CS nanoparticles, isolated hPAH tetramers were incubated with TPP solution at concentrations suitable to maintain the volume ratios of the blank nanoparticle formulations. Non-encapsulated protein was separated by centrifugation at 40,000× *g* for 30 min at 4 °C, after 30–60 min stabilization, at RT.

System optimization involved modulation of the initial pH of CS solution, CS and TPP concentration, presence of protein and protein loading capacity.

### 2.4. Particle Size and Zeta Potential

Mean particle size and polydispersity index (PI) were determined by photon correlation spectroscopy on a Zetasizer Nano-S (Malvern Instruments; Malvern, Worcestershire, UK) at a detection angle of 175° and a temperature of 25 °C with 2 min of stabilization time before measurements, samples were dispersed in purified filtered water (refraction index 1.330; viscosity 0.8872 cP, 25 °C), each sample was measured three times. Zeta potential was measured through laser Doppler anemometry on a Zetasizer Nano-Z (Malvern Instruments). Samples were diluted with 0.45 µm filtered ultra-pure water. In all cases, mean values were obtained from the analysis of three different batches, each of them measured three times. Results were expressed as mean ± standard deviation (SD).

### 2.5. Transmission Electron Microscopy (TEM)

The morphology of the nanoparticles was examined by transmission electron microscopy (TEM) on a H-8100 microscope (Hitachi High-Technologies Corporation, Tokyo, Japan). Nanoparticles were deposited in 200 mesh Cu formvar/carbon support film grids (Pelco^®^ Tem grid Support Films, Ted Pella, Inc., Redding, CA, USA), dried for 10 min and further analyzed.

### 2.6. Nanoparticle Yield

Nanoparticle production was assessed either by the intensity of diffracted light determined by photon correlation spectroscopy (Zetasizer Nano-S, Malvern Instruments), or by an indirect colorimetric quantification of the CS present in the supernatant after nanoparticle deposition by centrifugation. The latter methodology is based on the reaction between CS amine groups and the dye Cibacron brilliant red 3B-A, adapted to a 96-well microplate [30]. Briefly, nanoparticle suspension was centrifuged (Alegra™64R Centrifuge, Beckman Coulter, Brea, CA, USA) at 40,000× *g* for 30 min at 4 °C and after addition of the dye to the supernatant, absorbance was measured at 575 nm in a multi-mode microplate reader (FLUOstar Omega, BMG Labtech GMBH, Ortenberg, Germany). A standard curve was created using a known range of CS concentrations in the same conditions as the unknown samples. The percentage of CS incorporation was determined using Equation (1), where [CS]_total_ is the concentration of CS used to prepare the nanoparticles and [CS]_sup_ the measured concentration of free CS present in the supernatant.

CS incorporation (%) = ([CS]_total_ − [CS]^sup^)/([CS]_total_) × 100 (1)

### 2.7. Protein Quantification

The concentration of purified fusion proteins was measured considering that hPAH solution at 1 mg/mL presents an absorption of 0.91 (at λ = 280 nm).

The percentage of protein encapsulation was quantified indirectly using the BCA protein assay (Thermo Fisher Scientific, Rockford, IL, USA) to determine the protein concentration in the supernatant after nanoparticle deposition (as above). All samples were analyzed in triplicate. Encapsulation efficiency (EE) was determined using Equation (2), where [hPAH]_total_ represents the total amount of hPAH added to each sample and [hPAH]_sup_ the amount of hPAH present in the supernatant after nanoparticle deposition.

EE (%) = ([hPAH]_total_ − [hPAH]_sup_)/([hPAH]_total_) × 100 (2)

### 2.8. Enzymatic Assay

The hPAH activity was measured as previously described [29]. The reaction mixture contained 1 mM l-Phe, 100 mM Na-Hepes, pH 7.0, 0.1 mg/mL catalase, 5 μg of recombinant hPAH (non-encapsulated or encapsulated), 5 mM dithiothreitol DTT and 100 μM ferrous ammonium sulphate, in a final volume of 200 μL. Unless otherwise stated, after 4 min incubation at 25 °C, the reaction was started by the addition of 75 μM BH_4_ (pre-activated condition). The amount of L-Tyr produced after 1 min was quantitated by HPLC using a LiChroCART^®^ 250-4 LiChrospher^®^ 60 RP-select B (5 μm) column (Merck KGaA, Darnstadt, Germany), a 5% EtOH mobile phase pumped at 0.7 mL/min flow rate and fluorimetric detection (λ_exc_ = 274 nm and λ_em_ = 304 nm) [31]. To study the effect of pre-incubation with l-Phe on the specific activity, 1 mM l-Phe was added together with 75 µM BH_4_ at the initiation of the hydroxylation reaction (non-activated condition). Adequate controls consisting of empty nanoparticles were also performed.

### 2.9. Differential Scanning Fluorimetry (DSF)

Thermal unfolding profiles were obtained by DSF, in a C1000 Touch thermal cycler with a CFX96 optical reaction module (Bio-Rad; Hercules, CA, USA). All assays were carried out in SEC buffer at a final hPAH concentration of 100 µg/mL (non-encapsulated or encapsulated), in SEC buffer and with Sypro Orange at a 2.5× final concentration. PCR plates were sealed with Optical-Quality Sealing Tape (Bio-Rad) and centrifuged at 500× *g* for 5 min. Thermal profiles were obtained by ramping the temperature between 20 and 90 °C at 1 °C/min, with a 1 s hold time every 0.2 °C, and fluorescence acquisition through the FRET channel. Data were analyzed with CFX Manager Software V3.0 (Bio-Rad) and GraphPad Prism software V6.00 (La Jolla, CA, USA), fitting the experimental curves with a biphasic dose-response function to obtain the midpoint of the first and second transitions, which correspond to the melting temperatures (*T*_m_) of the regulatory (*T*_m1_) and catalytic domains (*T*_m2_). Adequate controls consisting of empty nanoparticles were also performed.

Differential scanning fluorimetry was also used to assess the l-Phe apparent binding affinity for hPAH. In this assay, the enzyme was incubated with increasing concentrations of l-Phe (0–22 mM) and the shifts in *T*_m1_ and *T*_m2_ rendered the concentration for half-maximal l-Phe binding (*C*_0.5_) for the regulatory (*C*_0.5(1)_) and catalytic (*C*_0.5(2)_) domains [32].

### 2.10. In Silico Modelling

The 3D representation of single and 50 grouped, charged and neutral CS polymeric chains was generated in Materials Studio 7.0 (Accelrys, Inc., San Diego, CA, USA) according to the described DD (91.8%). A small CS oligomer of 11 residues of β-(1-4)-linked D-glucosamine and one *N*-acetyl-d-glucosamine was replicated 28, 66 and 104 times to cover the distribution of CS MW from 50 to 120 and up to 190 kDa. For the CS oligomer, TPP and glucosamine, the logD was determined as this parameter describes the lipophilicity of a molecule taking into consideration its charge. The distribution of protonated microspecies of TPP and glucosamine throughout pH were also estimated. The in silico studies were performed with MarvinSketch V6.2.0 physicochemical property predictors (ChemAxon, Budapest, Hungary).

To analyze the impact of the nanoformulation on hPAH structure, a composite full-length model was generated from the truncated forms of rat PAH (pdb ID: 1PHZ) and human PAH (pdb ID: 2PAH) using the UCSF Chimera (University of California, San Francisco, CA, USA) [33]. Hydrophilic surface depiction was created with the solvent-excluded molecular surfaces MSMS package [34]. Protein protonation, throughout the tested pH range, was computed in UCSF Chimera with PROPKA and PDB2PQR using the optimized forcefield for Poisson-Boltzmann calculations PEOEPB [35,36,37,38,39]. Electrostatic surface potential was computed with the generated PQR files in the Adaptive Poisson-Boltzmann Solver (APBS) [40]. The hPAH free energy of folding throughout the pH was computed with the two pdb files used in the composite of the full-length hPAH (2PAH and 1PHZ) with the Bluues server for electrostatic properties of proteins based on generalized Born radii [41].

The hPAH:CS weight ratio (*w:w*) was further evaluated with molecular docking of the full-length composite PQR file charged at the final formulation pH with charged CS chains of 120 kDa (average MW) in the PatchDock server [42]. For each docking assay, the best 1000 hits were refined with FireDock server and sorted according to the minimum global energy (attractive/repulsive van der Waals, atomic contact energy and hydrogen bonding) [43]. The hits with the lowest global energy were overlapped according to the selected hPAH:CS (*w*:*w*) ratios.

### 2.11. Controls and Statistical Analysis

Adequate controls for the described assays consisted of the non-encapsulated hPAH tetramer in solutions containing the studied components at the tested concentrations (SEC buffer, TPP or CS). Data from independent experiments (*n* ≥ 3) are shown as mean ± SD. When applied, statistical significance (*p*) was determined by the Student’s paired *t*-test and compared the hPAH with the corresponding control sample (*p* < 0.05 was considered significant). For the thermal denaturation assays, a significant change in *T*_m_ was considered when |ΔT_m_| > 2 °C [44]. Where applicable, non-parametric analysis was performed using the Kruskal–Wallis test, followed by multiple comparisons using Dunn’s tests.

## 3. Results and Discussion

### 3.1. Protonation Degree and Polymeric Conformation

**Figure 1:**
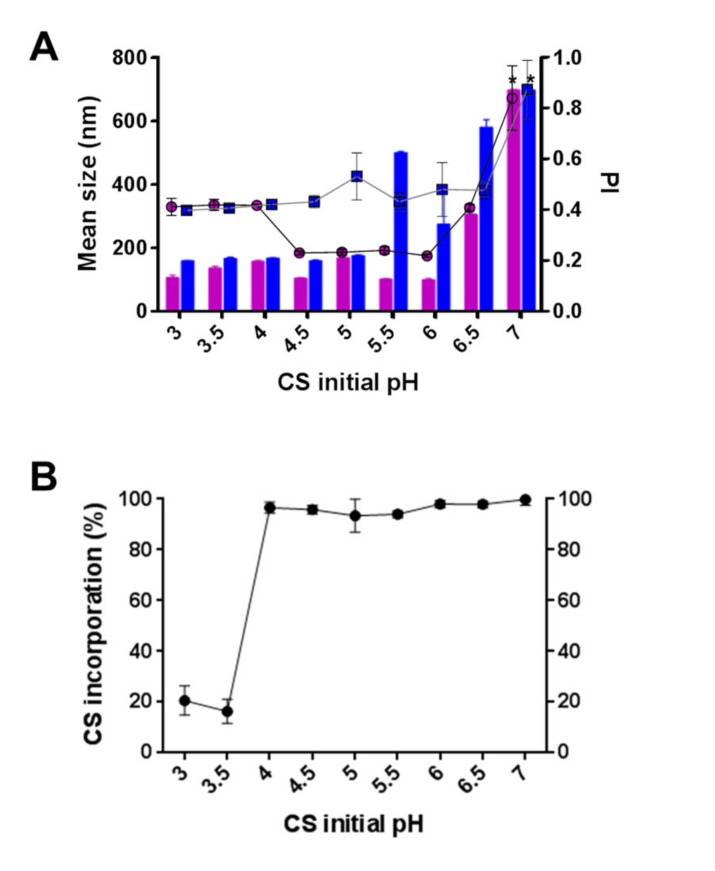
Effect of chitosan (CS) initial pH on the characteristics of empty and human phenylalanine hydroxylase (hPAH) loaded nanoparticles. (**A**) Mean size (bars) and polydispersity index (PI) (lines) of empty (pink circles) and hPAH loaded nanoparticles (blue squares) as monitored by dynamic light scattering (DLS); * indicates aggregated samples. (**B**) CS consumption during empty particle formation as measured by cibacron brilliant red 3B-A staining. Values represent mean ± S.D. (*n* = 3). Nanoparticles were obtained at a final concentration of CS at 833 µg/mL, tripolyphosphate (TPP) at 333 µg/mL and hPAH at 55 µg/mL (2.5:1 CS:TPP and 15:1 CS:hPAH *w*:*w* ratio). For further details see Materials and Methods.

Chitosan is a natural cationic polymer derived from the N-deacetylation of chitin, comprised of β-(1-4)-2-amino-2-deoxy-d-glucopyranose and a varying amount of β-(1-4)-2-acetamino-2-deoxy-d-glucopyranose residues according to its DD. The hydrodynamic properties of this polymer are strongly modulated by its MW and DD, which control both viscosity and the number of free amines (pKa of 6.5), and by the surrounding pH, which strongly impacts their ionization status. Taking into account that the purified hPAH solution is at pH 7 and the addition of 1% acetic acid to solubilize the CS stock solution renders a pH of 4 in the final CS solution, the behavior of the particulate system as a function of the CS initial pH value was assessed both with empty and hPAH-loaded CS-TPP particles (Figure 1). For pH modulation studies, the CS:TPP weight ratio (*w*/*w*) was initially fixed at 2.5:1 (rendering a final concentration of CS at 833 µg/mL and TPP at 333 µg/mL). After the addition of the buffered counterion either with or without hPAH (at pH 7.0), the final formulation pH increased by a factor of 0.5 throughout the tested range.

**Figure 2:**
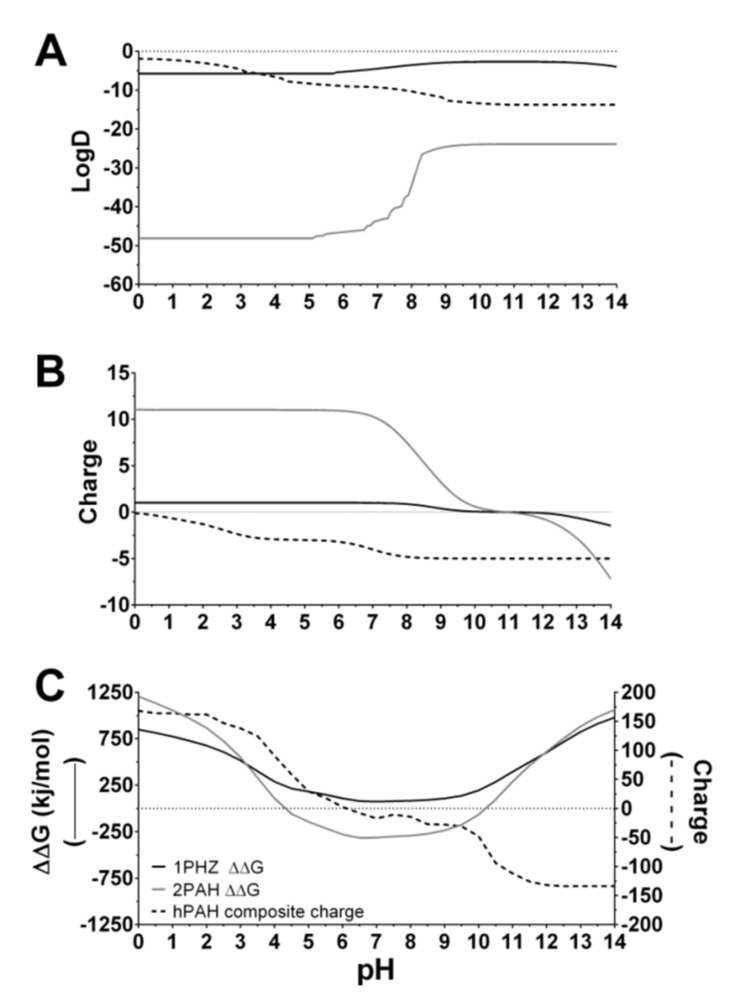
In silico prediction of the pH-dependent characteristics of nanoparticle constituents. (**A**) Distribution coefficient (logD) and (**B**) total charge of glucosamine (—), tripolyphosphate (TPP) (--) and chitosan (CS) oligomer (—) used to generate the extended polymeric chains with a deacetylation degree (DD) of 91.8%. (**C**) Free energy of human phenylalanine hydroxylase (hPAH) folding obtained from the pdb files used to generate the full-length composite—1PHZ (—) and 2PAH (—); Total charge of the full-length hPAH composite (--). For further details see Materials and Methods.

As previously reported, the ionic gelation of CS was found to be strongly modulated by the pH [45]. As the pH decreased from 4.5 to 3.0, the ionic gelation of CS with TPP empty particles was hampered, with a marked PI increase (≥0.4) and low CS incorporation (≈20%) (Figure 1A,B). In silico analysis correlates this behavior mainly with the protonation degree of TPP, as from above pH 4.5 its evenly distributed triple charge is reduced to neutralization through four different ionic species (Figure 2B and Figure S3). Such behavior hinders both its solubility (increased logD value) and the ability to interact with the positively charged CS polymeric chains (Figure 2A,B).

The TPP’s protonation degree impact on the ionic gelation of CS correlates with the results of Shu and Zhu, whereby an increasing amount of negative charges from sulphate, citrate and tripolyphosphate conferred an increasing physical resistance and a delayed drug release profile of CS beads [46]. From pH 4.5 to 6 (below the 6.5 pKa of CS amine groups), empty particles were produced with greater control of particle size (≤150 nm), PI (≤0.25) and CS incorporation (≥95%) (Figure 1A,B). Within this range, a finely tuned balance was achieved between TPP’s evenly distributed triple charges and the still protonated glucosamine residues (Figure 2B, Figures S3 and S4). Additionally, although there is no visible variation in the protonation status of the amine groups, CS solubility starts to decrease (increased logD; Figure 2A).

**Figure 3:**
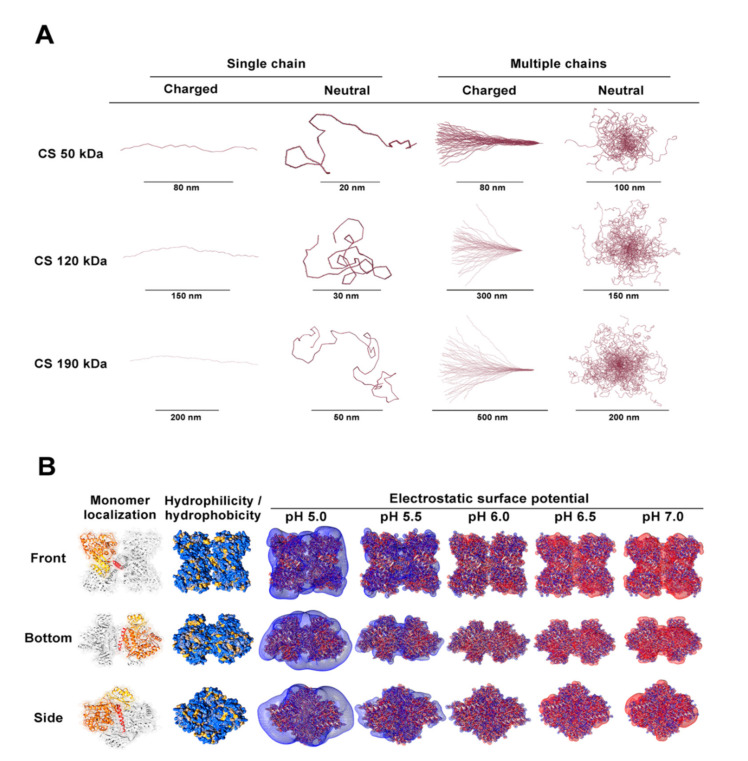
In silico assessment of chitosan (CS) hydrodynamics and human phenylalanine hydroxylase (hPAH) hydrophilicity and electrostatic surface potential at different pH values. (**A**) Hydrodynamic behavior of charged/neutral CS polymeric single and 50 chains at the minimum (50 kDa), maximum (190 kDa) and average (120 kDa) CS MW used in this study. (**B**) In silico evaluation of hydrophilicity/hydrophobicity and electrostatic potential of tetrameric hPAH; (Monomer localization) regulatory (yellow), catalytic (orange) and oligomerization (red) domain of a monomer; (hydrophilicity/hydrophobicity) hydrophilic (blue) and hydrophobic (yellow) hPAH residues; (Electrostatic surface potential) positive surface potential (blue) and negative surface potential (red). For further details see Materials and Methods.

In a diluted solution, low MW CS with a strong DD has been reported to attain an extended linear conformation due to the strong intra and intermolecular electrostatic interactions and hydrogen bonding [45,47,48]. As the distribution of charges is shifted and the electrostatic repulsion is reduced, the conformation of CS polymeric chains is changed into a random coil [45,47,48]. Such behavior was in agreement with the in silico data generated from the specific polymer’s MW and DD used in this study, correlating the equilibrium between the two states with the optimum balance for CS to interact in a controlled manner with TPP (Figure 1A and Figure 3A).

As the initial value of CS pH surpassed the pKa value for the amine groups (i.e., 6.5), neutralization of the polymer produced a clear destabilization of the colloidal system with a marked increase of PI (≥0.4) and particle size up to visible aggregation (Figure 1A). From this point on, both CS protonation and solubility decreased markedly and the increased protonation of TPP contributed to an entropic aggregation process.

Within the optimal pH range found for empty nanoparticle formulation (i.e., 4.5–6.0), inclusion of the enzyme at a final concentration of 55 µg/mL corresponding to a CS:hPAH ratio of 15:1 (*w*:*w*) destabilized the system with an unwanted increase of particle size (≥300 nm) and PI (≥0.4) (Figure 1A). The hPAH entropic contribution to the system (complex quaternary structure and hydrophilic surface) correlates with the in silico prediction of its average charge and electrostatic potential throughout the pH range. At the formulation pH (7.0), the enzyme presents a mild negative average charge (Figure 2C) with a general negative electrostatic surface potential (Figure 3B), thus acting as a counter-ion during its entrapment in the CS-TPP network and exceeding the previously found optimized equilibrium.

Therefore, taking into consideration the optimum initial CS pH range for empty nanoparticle formulation (4.5–6.0), and the minimum hPAH free energy of folding ranging from pH 6.0 to 7.0 (Figure 2C), to avoid a denaturing stress and achieve a compatible biological formulation, initial CS pH values of 5.5 and 6.0 were selected for further optimization of the colloidal system.

### 3.2. Characterization of CS-TPP Interplay

**Figure 4:**
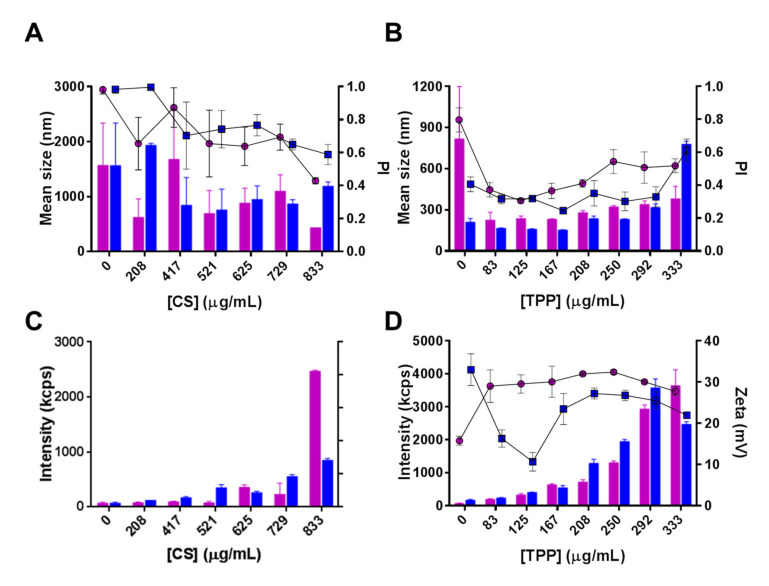
Effect of chitosan (CS) and tripolyphosphate (TPP) concentration on the characteristics of nanoparticles loaded with human phenylalanine hydroxylase (hPAH) at a final concentration of 55 µg/mL. (**A**,**B**) Mean size (bars) and PI (lines) at pH 5.5 (pink) and 6.0 (blue), as monitored by DLS. (**C**,**D**) Nanoparticle yield by intensity of diffracted light by dynamic light scattering (DLS) (bars) and zeta potential (lines) at pH 5.5 (pink) and 6.0 (blue). Values represent mean ± S.D (*n* = 3). For further details see Materials and Methods.

As shown in Figure 4A, an initial CS:hPAH ratio of 15:1 (*w:w*) and CS:TPP of 2.5:1 (*w:w*), resulted in the formation of large, polydispersed particles (≥400 nm; PI ≥ 0.4), irrespective of the tested pH values. As the concentration of CS diminished from 833 µg/mL, a marked decrease in particle yield, as monitored by the intensity of diffracted light in DLS, was also observed, due to a decreased CS protonation with a reduced ionic gelation at pH 6.0 (Figure 4A,C). At both pH conditions (5.5 and 6.0), a decreasing the amount of TPP added to the system (at a constant 833 µg/mL CS and a CS:hPAH ratio of 15:1) resulted in an increased control of the ionic gelation process up to optimal concentration values (125 µg/mL TPP at pH 5.5 and 167 µg/mL TPP at pH 6.0) from which destabilization once more occurred (Figure 4B). Several parameters took part in this interplay. The optimized controlled condition (pH 6.0 at TPP 167 µg/mL; Figure 4B) that produced nanoparticles presenting lower particle sizes and a narrower distribution, was preceded by a zeta potential minimum (pH 6.0 at TPP 125.0 µg/mL; Figure 4D) that pinpoints the molecular trigger from which the conformational rearrangement of polymeric random coil packed chains undergo a controlled assembly into nanoparticles (Figure 3A) [45,47,48]. As an increasing number of CS polymeric chains are packed into one another, a simultaneous increase in particle size, yield and surface charge is observed (Figure 4B,D).

At pH 5.5 such a minimum is achieved at lower concentrations as the increased protonation and solubility of CS enhances its interaction with TPP and hPAH (Figure 2A,B). Furthermore, at pH 5.5, a decreased number of negative electrostatic pockets at the surface of hPAH (Figure 3B), as well as a slight decrease of TPP protonated species (Figure 2B and Figure S4), contribute to the overall less ordered interaction between CS and hPAH/TPP with a larger mean particle size, a higher PI and higher zeta potential values. Such events stresses out how the hydrodynamic properties of CS, hPAH and the optimized decrease of negative charges from TPP play a key role in the ionic gelation process.

Hence, the optimized process conditions were obtained at pH 6.0 and a TPP concentrations of 167 µg/mL, resulting in lower mean particle size (≈150 nm) and PI (≤0.25).

### 3.3. hPAH Loading, Protein Stability and Nanofunctionality

**Figure 5:**
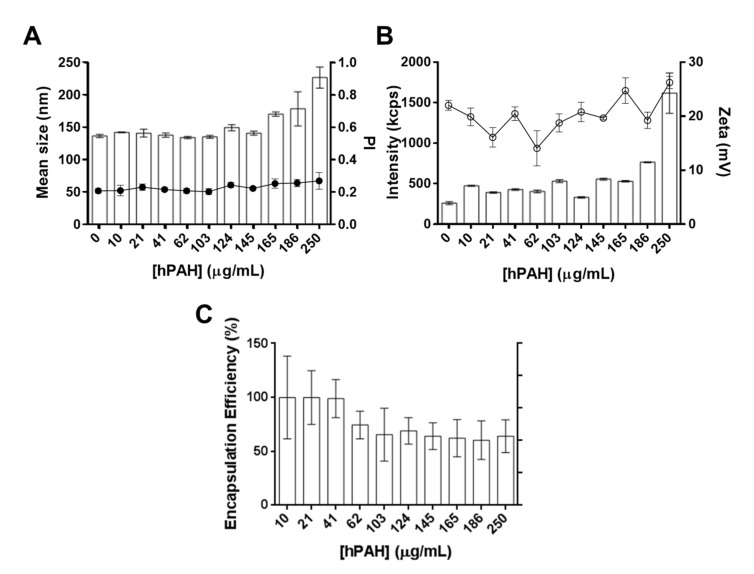
Effect of human phenylalanine hydroxylase (hPAH) loading on the characteristics of optimized nanoparticles (5:1; CS:TPP weight ratio). (**A**) Mean size (bars) and PI (lines) monitored by dynamic light scattering (DLS). (**B**) Nanoparticle yield by intensity of diffracted light (bars) and zeta potential (lines) by DLS. (**C**) hPAH encapsulation efficiency monitored by BCA protein assay; a non-parametrical analysis was performed as referred in Section 2.11 (Controls and Statistical Analysis). Values represent mean ± S.D (*n* = 3). For further details see Materials and Methods.

Using the previously optimized conditions (CS:TPP weight ratio of 5:1, initial CS pH 6.0), the system was further characterized regarding a hPAH loading of up to 250 µg/mL, which corresponded to a maximum CS:hPAH weight ratio of 3.3:1 (Figure 5).

**Figure 6:**
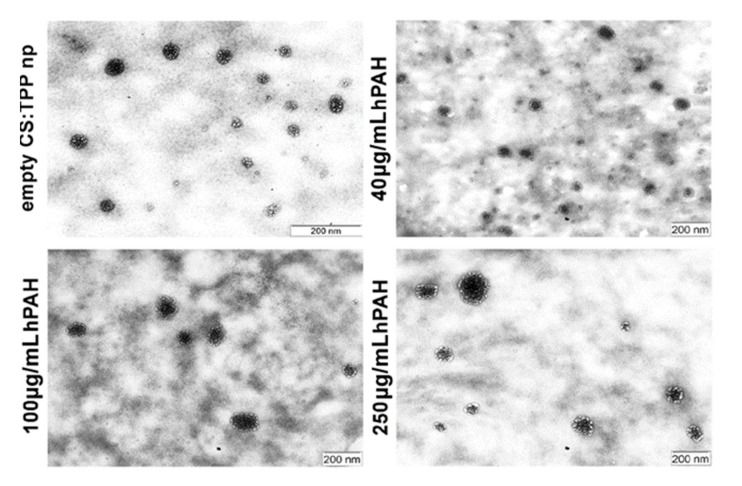
Transmission electron microscopy (TEM) analysis of optimized nanoparticles (5:1; CS:TPP weight ratio) with different hPAH loading. For further details see Materials and Methods.

Throughout the tested range of enzyme concentrations, the nanoparticulate system was able to entrap hPAH without a severe impact on the formulation main physicochemical properties. Mean particle size and PI slightly increased from a hPAH loading of 100 µg/mL (CS:hPAH ratio of 8:1) without extensive PI destabilization (≈0.25) (Figure 5A). Surface charge and nanoparticle yield followed the same behavior as the negative electrostatic surface regions of the enzyme contributed to the gelation of the colloidal system (Figure 5B). While the CS solution was prepared at an initial pH value of 6.0, the pH of the final formulation was 6.5 after buffered hPAH and TPP were added. Considering that hPAH presents a mild negative average surface charge at the final formulation pH (Figure 2C and Figure 3B), the increase in nanoparticle zeta potential points to a gelation process where the enzyme is entrapped within the CS-TPP matrix as opposed to a surface protein adsorption model. Morphological analysis through TEM supported the light scattering measurements, showing a spherical homogeneous nanoparticle population with a similar mean particle size (Figure 6).

Results indicate that the developed system presents a robust ability to entrap hPAH without a substantial destabilization of the colloidal system. Although the mean values of the encapsulation efficiency (Figure 5C) show a decrease down to 65% when using an hPAH loading above 41 µg/mL (CS:hPAH ratio of 20:1), statistical analysis indicates that these differences were not statistically significant (*p* > 0.05). Using similar nanoencapsulation approaches, an EE lower than 55% has been described for the formulation of tetanus (MW ≈ 150 kDa) and diphtheria (MW ≈ 62 kDa) toxoids [7]. In addition, prolidase encapsulation in CS glutamate nanoparticles needed several harsh sonication steps, producing mean particle sizes ≥350 nm, a PI ≥0.4 and an EE ≤42% [49]. These reports emphasize how a careful and optimized rational design may dictate the viability of such difficult formulation processes and complex drug delivery systems.

**Figure 7:**
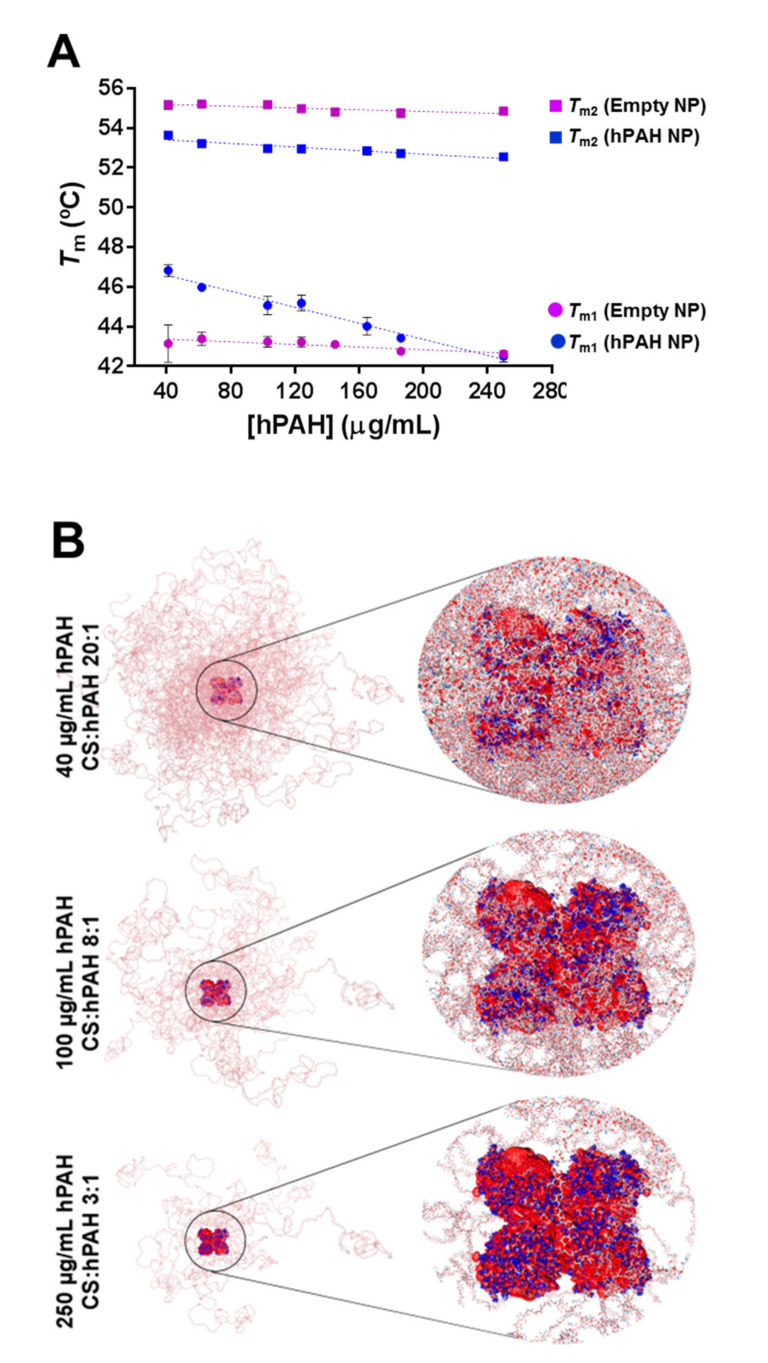
Effect of protein concentration on the thermal stability of the regulatory (*T*_m1_) and catalytic (*T*_m2_) domain of nanoencapsulated human phenylalanine hydroxylase (hPAH) and molecular crowding of nanoparticles. (**A**) Thermal stability of the regulatory (*T*_m1_; circle) and catalytic (*T*_m2_; square) domains of buffered (pink) and encapsulated hPAH (blue) monitored by differential scanning fluorimetry (DSF). (**B**) Depiction of the molecular crowding of encapsulated hPAH obtained by superposition of the docking hits with generated CS polymeric chains according to the selected loading ratios. The hPAH electrostatic surface potential at the final pH is depicted in blue (positive surface potential) and red (negative surface potential); values represent mean ± S.D. (*n* = 3). For further details see Materials and Methods.

The DSF technique successfully probed the thermal stability of the entrapped protein (Figure 7). Using this approach, the hPAH concentration, whereby the enzyme presented the maximum *T*_m_ for both the regulatory and catalytic domains (*T*_m1_ and *T*_m2_), was obtained at 40 µg/mL (CS:hPAH ratio of 20:1). Remarkably, when comparing both hPAH encapsulated and buffered thermal denaturation profiles, a clear and significant stabilization of the encapsulated regulatory domain is evidenced (*T*_m1_ ≥ +2 °C), as opposed to a slight and non-significant destabilization of the catalytic domain (*T*_m2_ ≈ −1 °C). These data suggest that the entrapment of the protein in the polymeric matrix contributes to the stabilization of the regulatory domain. In fact, studies by Agudelo et al. suggested that CS chains are able to bind both hydrophilic and hydrophobic moieties, inducing protein stabilization [50].

Since the N-terminal regulatory domain has been extensively described as one of the most relevant players affecting the hPAH aggregation-prone profile and functional regulation, an effective stabilization of this domain again stresses the valuable therapeutic potential of a CS-based system applied to such a complex enzyme [51]. As the hPAH-loading increased, both *T*_ms_ decreased and such an effect, as expected, was more prominent for the less stable regulatory domain (Figure 7A). However, even at the highest hPAH-loading tested (250 μg/mL), the obtained *T*_m1_ was similar to the value obtained for the buffered protein.

Using DSF, the ability of the nanoencapsulated hPAH to bind l-Phe (*C*_0.5_) was also investigated (Table 1 and Figure S5) within the range of the CS:hPAH weight ratio from 3:1 to 20:1 and compared to the buffered protein.

Interestingly, this parameter proved to be critical in the modulation of the functional behavior of hPAH. At 40 µg/mL, hPAH (20:1; CS:hPAH) significantly reduced the l-Phe apparent binding affinity for both the regulatory (2.7-fold) and catalytic (4.7-fold) domains. In silico molecular docking of CS polymeric chains superimposed with hPAH (Figure 7B) suggest that at this concentration (40 µg/mL hPAH; CS:hPAH ratio of 20:1), substrate diffusion and protein flexibility must be constrained as a dense matrix of CS polymeric chains surrounds the hPAH tetramers. A similar effect is observed in the induced compaction of proteins by natural osmolytes and when the electrostatic binding profile and solvation energetics of a protein is impacted by the surrounding molecular crowding [52,53]. Moreover, at 250 µg/mL hPAH (CS:hPAH ratio of 3:1), where the highest nanoparticle size and PI was obtained, although the apparent affinity towards the regulatory domain does not present significant variation (1.4-fold), the apparent affinity of the catalytic domain is again reduced (3.1-fold), suggesting that a change in the conformation of this domain may have occurred. A finely tuned balance is achieved at 100 µg/mL (CS:hPAH ratio of 8:1) where the balance between the CS polymeric crowding surrounding hPAH and the exposure to the solvent did not contribute to a change in the l-Phe apparent binding affinity, when compared to the buffered enzyme.

With an optimized CS-hPAH-TPP formulation at 100 µg/mL hPAH, the ability for hPAH to be activated by l-Phe pre-incubation and more importantly, the relative enzymatic activity, was further assessed (Table 1). Accordingly, enzymatic activity and substrate activation were maintained after encapsulation. In fact, when compared to the buffered enzyme, the nanoencapsulated protein maintained 100% residual activity and a l-Phe activation ratio of 2.89 (buffered hPAH: 2.78). Data indicate that the entrapped hPAH still presents the necessary flexibility to respond to l-Phe activation through the movement of the N-terminal regulatory domain.

## 4. Conclusions

Bridging the knowledge obtained by in silico and in vitro data, we were able to detect the critical parameters that impacted the ionic gelation of CS, TPP and hPAH and optimize a colloidal system that usually renders entropic large mean nanoparticle sizes and broader distributions. Moreover, the designed CS-based nanoparticulate system effectively delivers a complex multimeric therapeutic enzyme, with particle sizes ≤200 nm and higher EE than other previously reported entrapped therapeutic proteins. Having achieved an increased mechanistic comprehension on how each of the variables behaved within a complex network, a greater control in the ionic gelation of CS, hPAH and TPP rendered a reproducible, stable and self-contained entrapment method. Such a devised system proved to be effective in maintaining protein stability and enzymatic function. Hence, from a correct characterization of the CS, TPP and hPAH interactions, it is possible to provide a systematic approach to the nanoformulation of complex therapeutic enzymes using a mild and biocompatible ionic gelation method.

Taken together, the results here reported constitute a significant milestone towards the development of a potential novel therapeutic strategy to address diseases benefitting from an enzyme replacement therapy. Considering there is still a large unmet clinical need for metabolic inherited disorders, a controlled and systematic approach to polysaccharide delivery systems may present as an excellent strategy of drug delivery to fulfil the formulation of complex large therapeutic enzymes. The next objective is to determine the patterns of protein release and whether the nanoencapsulated hPAH do indeed provide preclinically relevant therapeutic effects in vivo.

## Figures and Tables

**Table 1:** Catalytic activity, l-Phe activation ratio and l-Phe apparent binding (*C*_0.5_) of human phenylalanine hydroxylase (hPAH) encapsulated in nanoparticles (NP) at different concentrations.

	l-Phe Apparent Binding (mM) ^a^		Relative Enzyme Activity (%) ^b^	l-Phe Activation Ratio
	*C*_0.5(1)_^c^	*C*_0.5(2)_^c^		
Buffered hPAH	0.32 ± 0.07	1.07 ± 0.20	100 ± 8.3	2.78
NP-hPAH (40 µg/mL)	0.85 ± 0.12 *	5.07 ± 0.62 **	n.d.	n.d.
NP-hPAH (100 µg/mL)	0.38 ± 0.04	1.38 ± 0.14	106.7 ± 2.6	2.89
NP-hPAH (250 µg/mL)	0.44 ± 0.07	3.38 ± 0.41 **	n.d.	n.d.

Notes: Control refers to buffered hPAH tetramers (naked enzyme) stored in liquid N_2_. Data are expressed as mean ± SD (*n* = 3). A non-parametrical analysis was performed as referred to in Section 2.11 (Controls and Statistical Analysis). The *p* value is indicated whenever a significant statistical difference towards the control was observed (<0.05) and correspond to * <0.001 and ** <0.0001. ^a^ Determined by differential scanning fluorimetry; ^b^ The relative enzyme activity was determined at standard conditions (1 mM l-Phe, 75 µM BH_4_, 25 °C); ^c^ Concentration for half-maximal binding (*C*_0.5_) for the regulatory (*C*_0.5(1)_) and catalytic (*C*_0.5(2)_) domains. (n.d.) not determined. For further details see Materials and Methods.

## Supplementary Materials

The following are available online at https://www.mdpi.com/1999-4923/13/3/329/s1, Figure S1: Oligomeric profile of human phenylalanine hydroxylase (hPAH) determined by size exclusion chromatography after protein purification by immobilized metal affinity chromatography (IMAC); Figure S2: Size exclusion chromatography (SEC) calibration curve; Figure S3: In silico prediction of the distribution of tripolyphosphate (TPP) ionic species (1 to 8) according to the pH; Figure S4: In silico prediction of the distribution of glucosamine ionic species (1 and 2) according to the pH; Figure S5: l-Phe-binding assay of the regulatory and catalytic domains of human phenylalanine hydroxylase (hPAH) encapsulated at 40 µg/mL, 100 µg/mL and 250 µg/mL, monitored by differential scanning fluorimetry (DSF).
