# Supplementary material for: In Silico and In Vitro Tailoring of a Chitosan Nanoformulation of a Human Metabolic Enzyme
Source: Pharmaceutics. 2021 Mar 4;13(3):329. doi: 10.3390/pharmaceutics13030329 (PMC8000282; doi:10.3390/pharmaceutics13030329)
Supplement: Supplementary file 1 [file pharmaceutics-13-00329-s001.pdf]

pharmaceutics

Supplementary Materials: In Silico and In Vitro Tailoring of a Chitosan Nanoformulation of a Human Metabolic Enzyme

Paulo R. Lino, João Leandro, Mariana Amaro, Lídia M. D. Gonçalves, Paula Leandro and António J. Almeida

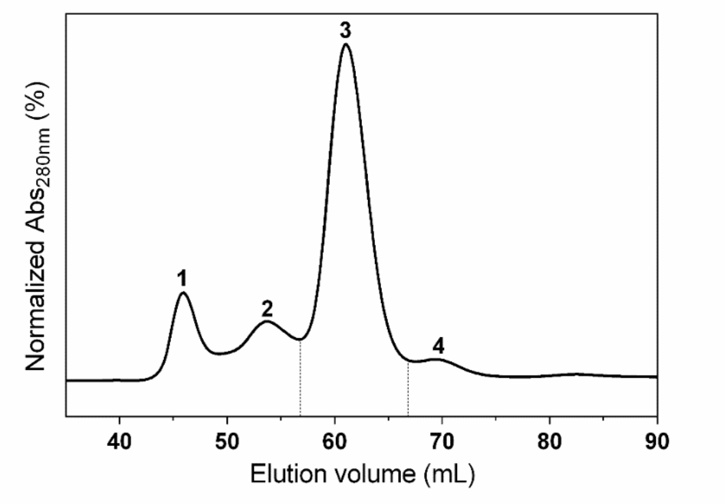

Figure S1. Oligomeric profile of human phenylalanine hydroxylase (hPAH) determined by size exclusion chromatography after protein purification by immobilized metal affinity chromatography (IMAC). Peak 1 represents higher-order oligomeric forms (eluted in the void volume; 10.7%), peak 2 a presumably octameric form (22.4%), peak 3 the tetramer (~ 220 kDa; 60.4%) and peak 4 the dimer (~ 110 kDa; 6.5%). The apparent molecular mass of the enzyme forms were estimated using the elution position of standard molecular mass markers as a reference (calibration curve presented in Supplementary Figure S2). Dashed lines indicate the interval of collected tetramers utilized for the nanoencapsulation assays. For further details see Materials and Methods.

Publisher's Note: MDPI stays neutral with regard to jurisdictional claims in published maps and institutional affiliations.

Copyright: © 2020 by the authors. Submitted for possible open access publication under the terms and conditions of the Creative Commons Attribution (CC BY) license (http://creativecommons.org/licenses/by/4.0/).

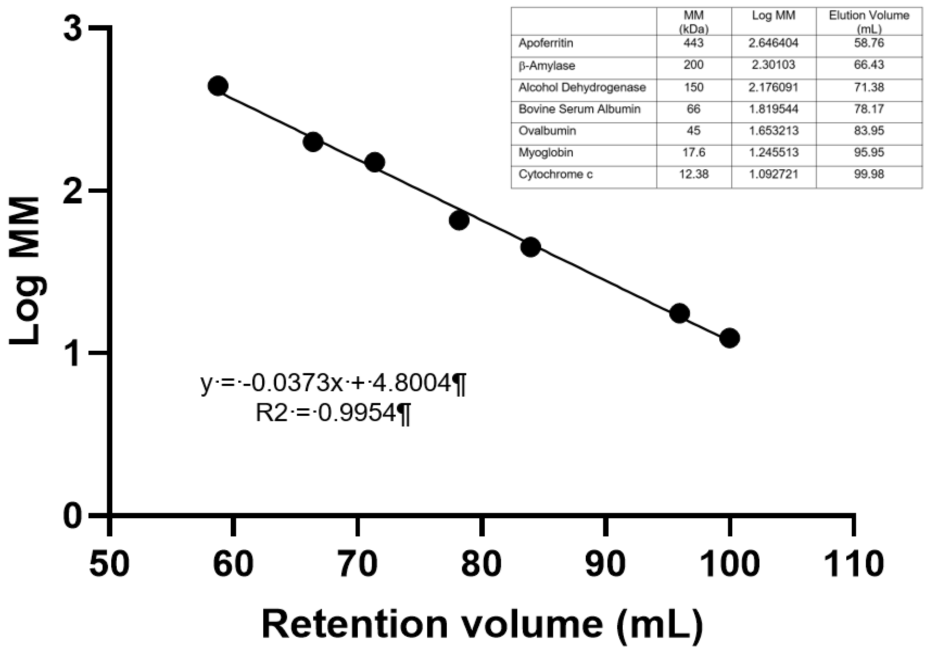

Figure S2. Size exclusion chromatography (SEC) calibration curve. The curve was utilized to estimate the molecular mass of the different oligomeric forms and consisted of the standard proteins: cytochrome C (12.4 kDa), myoglobin (17.6 kDa), ovalbumin (45 kDa), BSA (66 kDa), alcohol dehydrogenase (150 kDa), β-amylase (200 kDa) and apoferritin (443 kDa). The calibration curve equation is shown in the graph.

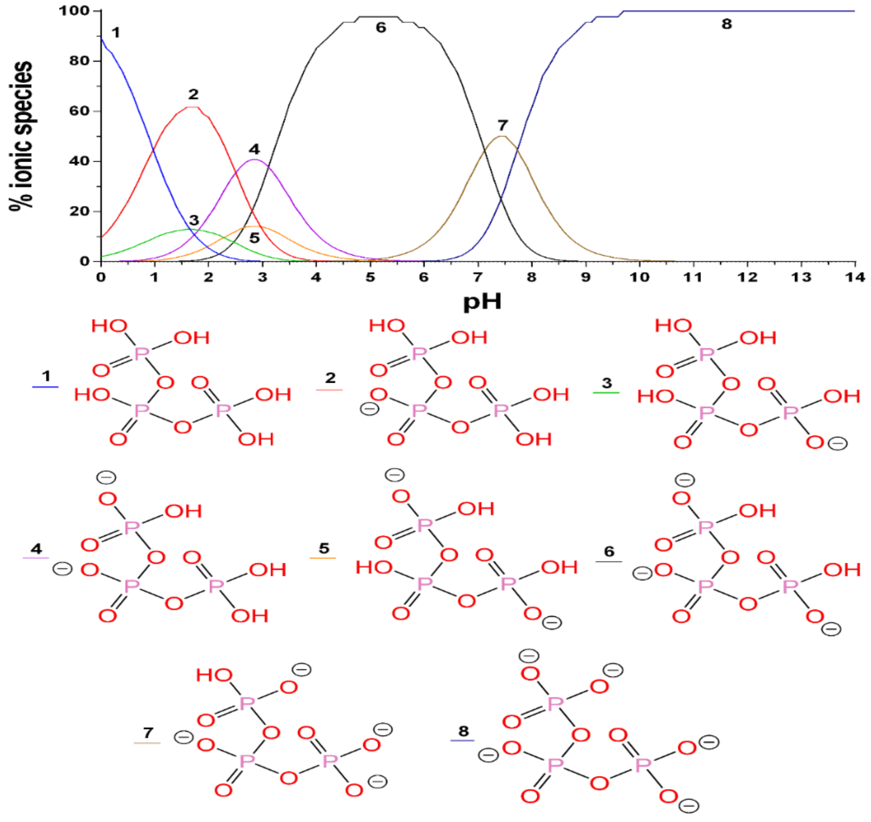

Figure S3. In silico prediction of the distribution of tripolyphosphate (TPP) ionic species (1 to 8) according to the pH. For further details see Materials and Methods.

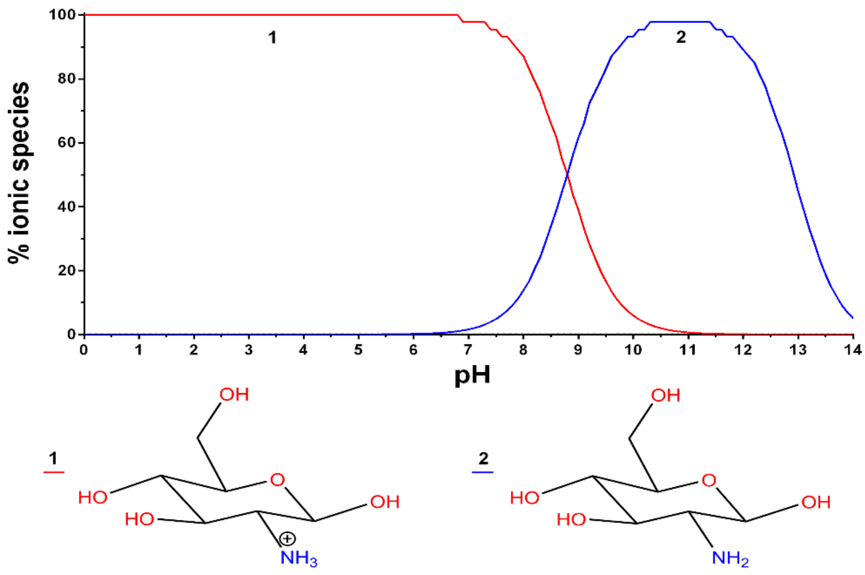

Figure S4. In silico prediction of the distribution of glucosamine ionic species (1 and 2) according to the pH. For further details see Materials and Methods.

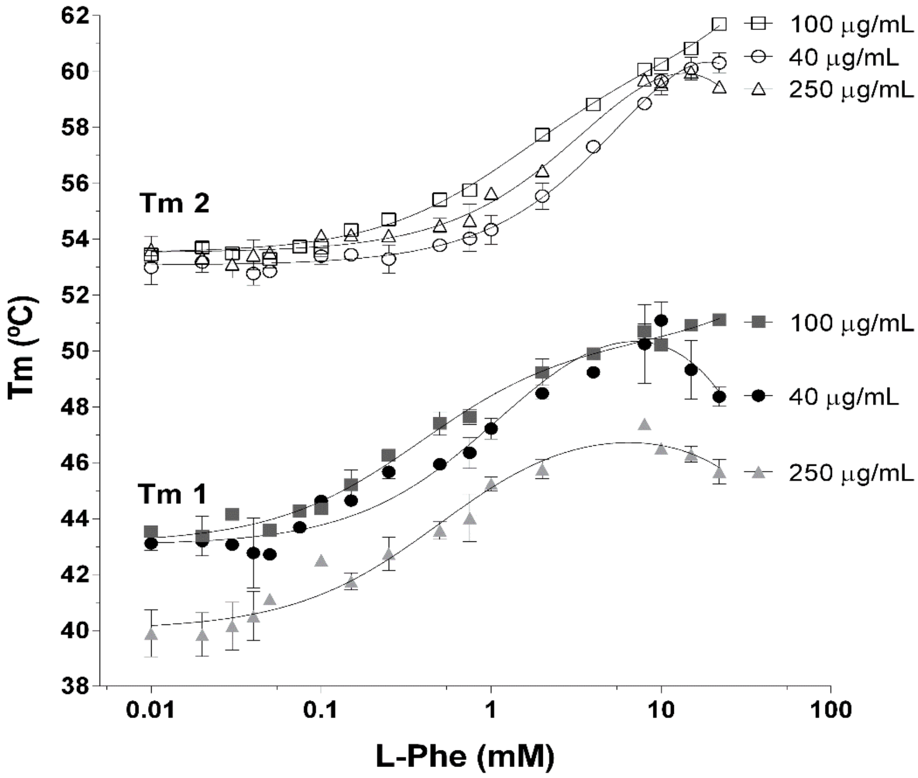

Figure S5. L-Phe-binding assay of the regulatory (full) and catalytic (empty) domains of human phenylalanine hydroxylase (hPAH) encapsulated at 40 µg/mL (circles), 100 µg/mL (squares) and 250 µg/mL (triangles), monitored by differential scanning fluorimetry (DSF). Values represent mean ± SD (n = 3). For further details see Materials and Methods.
